# Trichoepithelioma and tattooing: a causal or casual relationship?

**DOI:** 10.1111/srt.13474

**Published:** 2023-09-21

**Authors:** Cristian Fidanzi, Nicolò Mori, Matteo Bevilacqua, Salvatore Panduri, Marco Romanelli, Agata Janowska

**Affiliations:** ^1^ Unit of Dermatology University of Pisa Pisa Italy

Dear Editor,

Tattooing is an ancient art form that entails creating permanent skin designs by inserting external dyes into the dermis.^1^ This practice is increasingly popular, with approximately 25 percent of Americans and 10 percent of Europeans and Australians having at least one tattoo.^2^ Despite the procedure being conducted by professionals, there are several hypothesized complications mentioned in the literature, including inflammatory, infectious, and neoplastic issues.^1^, ^3^ However, a definite connection between tattoos and skin cancers has not been firmly established yet^1^

Trichoepithelioma is a rare benign tumor of the skin that originates from the hair follicle and has the appearance of a translucent papule of a few millimeters in size.^4^ It is typically present in autosomal dominant inherited syndromes, such as familial cylindromatosis or Brooke‐Spiegler syndrome which are associated with mutations in the CYLD gene^3^ In individuals affected by these syndromes, multiple lesions appear during childhood and adolescence, primarily localized on the neckline, face, and scalp.^4^ Occasionally, trichoepithelioma may sporadically occur in individuals over 40 years old, usually appearing on the face or scalp as one or a few lesions.^4^

We describe the case of a 45‐year‐old man who presented to our outpatient clinic for a nevi exam. During the clinical examination of the skin, we identified a pinkish‐yellowish papule measuring approximately 5 mm in size located on the tattooed region of the right shoulder (Figure 1). The tattoo had been done 10 years ago. Since the patient was unaware of the lesion, he was unable to provide any information on when it had occurred. On dermoscopic examination, shiny‐white lines, milia‐like cysts, a small central erosion and a peripheral bluish veil referable to the tattoo pigment were appreciated (Figure 2). Due to suspicions of a small basal cell carcinoma, we opted to surgically excise the lesion and perform a histological examination. The results, however, revealed a trichoepithelioma instead. Given the benignity of the lesion, no further treatment was necessary.

**FIGURE 1:**
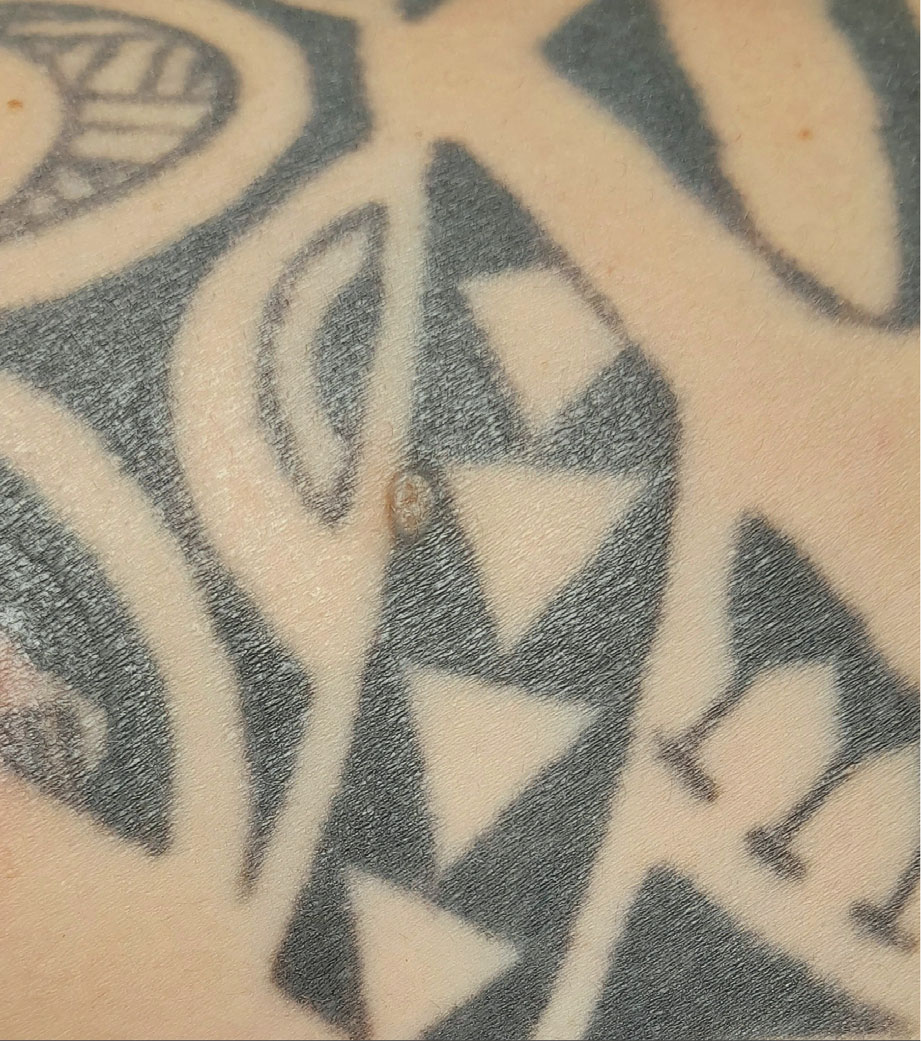
The clinical presentation of the lesion is that of a pinkish‐yellowish papule measuring approximately 5 mm in size, located on the right shoulder.

**FIGURE 2:**
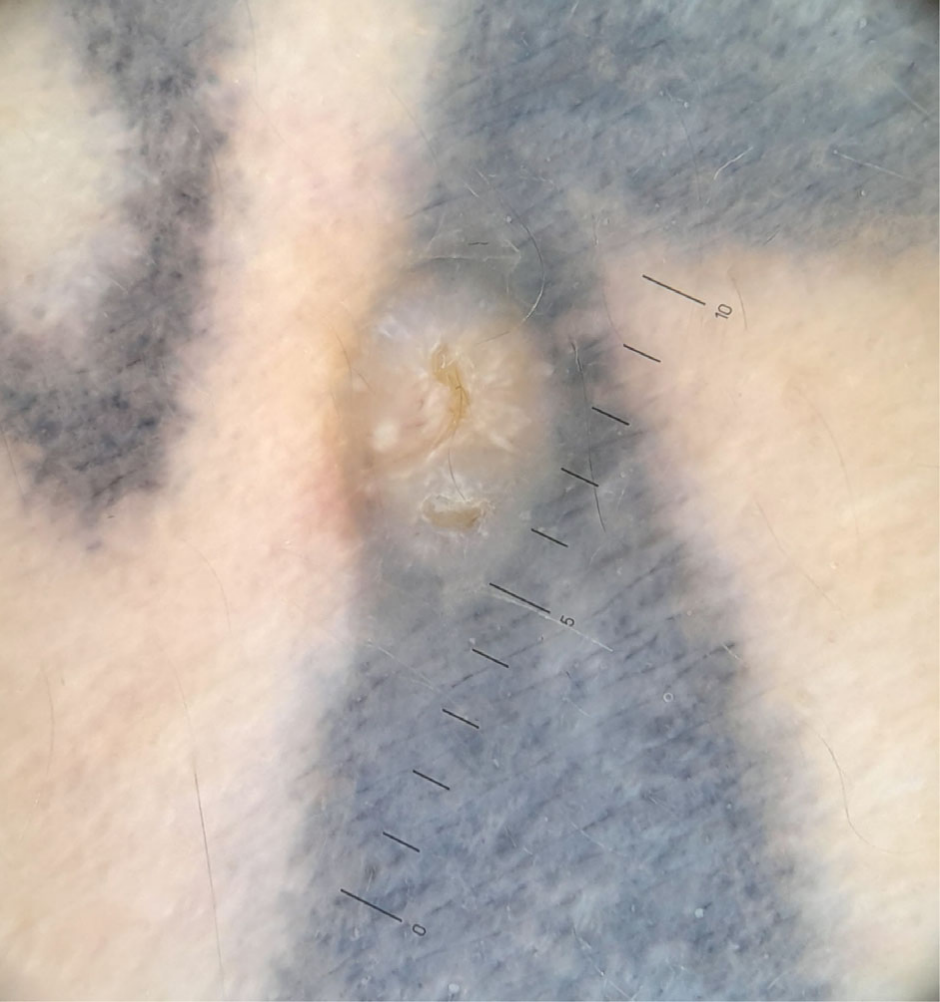
On dermoscopic examination shiny‐white lines, milia‐like cysts, a small central erosion and a peripheral bluish veiling referable to the tattoo's pigment can be appreciated.

To the best of our knowledge, there have been no documented cases of a trichoepithelioma occurring within a tattooed area until now. While we cannot completely rule out the possibility that this association was coincidental, the fact that it appeared at an unusual location (the arm) and at a younger age than sporadic lesions usually develop, raises suspicion about the potential role of tattoo pigment in transforming follicle cells.

The link between tattooing and the development of skin tumors is controversial.^1^ Tattoo pigments typically consist of inorganic metal salts and other organic molecules such or polycyclic compounds that have been classified as possibly carcinogenic to humans (Group 2B) and carcinogenic to humans (Group 1) by the International Agency for Research on Cancer.^5^ Moreover, exposure to UV rays from solar radiation may contribute to the breakdown of pigments into other potentially carcinogenic substances.^1^

Some authors have suggested that both acute trauma (needle penetration) and chronic irritation caused by tattoo pigments might promote cell proliferation, potentially leading to tumor development.^1^ However, despite the widespread prevalence of tattoos in industrialized countries, the actual number of observed neoplasms is relatively low, although it is likely underestimated.^2^ It is crucial to ensure that all new cases of tumors encountered in clinical practice are reported to tumor registries, as this can provide valuable insights into the potential oncogenic role of tattoos.^6^, ^7^ Trichoepithelioma is a benign tumor, but its identification and removal remain important due to the challenges in distinguishing it from certain malignant tumors and the possibility of progressing into basal cell or trichoblastic carcinomas^4^

## CONSENT STATEMENT

The patients in this manuscript have given written informed consent to publication of their case details

## Data Availability

The data that support the findings of this study are openly available in CF at https://pubmed.ncbi.nlm.nih.gov/37279633/.
